# Choroidal and Retinal Permeability Changes in Chronic Kidney Disease—A Literature Review

**DOI:** 10.3390/jcm14248767

**Published:** 2025-12-11

**Authors:** Giacomo De Rosa, Francesco Paolo De Rosa, Giovanni Ottonelli, Mario R. Romano

**Affiliations:** 1 Department of Biomedical Sciences, Humanitas University, Via Rita Levi, Montalcini 4, 20072 Pieve Emanuele, MI, Italy; 2 Eye Center, Humanitas Gavazzeni-Castelli, 24128 Bergamo, BG, Italy

**Keywords:** chronic kidney disease, hemodialysis, peritoneal dialysis, retinal disorders, choroidal thickness, retinal pigment epithelium, blood-retinal barrier

## Abstract

**Purpose**: This review consolidates current evidence on how chronic kidney disease (CKD)-especially end-stage kidney disease (ESKD) and its treatments-alters choroidal and retinal vascular permeability, leading to changes in intraocular fluid homeostasis. **Methods**: A literature search of Medical Literature Analysis and Retrieval System Online (MEDLINE), reference lists, and key ophthalmology-nephrology texts was performed for studies published between 1980 and 2025. One-hundred-forty-four articles (clinical trials, observational cohorts, and case reports) met the inclusion criteria. Data were abstracted on choroidal thickness changes, blood-retinal barrier integrity, incidence of Central Serous Chororioretinopathy (CSCR) and Serous Retinal Detachment (SRD) in dialysis and transplant populations, and systemic variables such as oncotic pressure, hypertension, and corticosteroid exposure, with special attention to retinal pigment epithelium (RPE) pump function. Findings were synthesized qualitatively and tabulated where appropriate. **Results**: ESKD induces a triad of lowered plasma oncotic pressure, fluctuating hydrostatic forces, and impaired RPE pump function that collectively drive subretinal fluid accumulation. Hemodialysis acutely reduces sub-foveal choroidal thickness by a mean of ≈15–25 µm yet shows inconsistent effects on retinal thickness. Large population data demonstrate a three- to four-fold higher SRD risk and ~1.5-fold higher CSCR risk in dialysis patients versus controls, with peritoneal dialysis conferring the greatest hazard. After kidney transplantation, CSCR prevalence approaches 6%, driven by combined stresses of surgery, hypertension, and long-term corticosteroid or calcineurin-inhibitor therapy. Most reported SRDs resolve as systemic parameters normalize, underscoring the importance of promptly identifying systemic drivers. **Conclusions**: Systemic fluid-pressure imbalances and treatment-related factors in CKD significantly perturb the outer blood-retinal barrier. Regular ophthalmic surveillance, early visual-symptom screening (e.g., Amsler grid), and close nephrologist-ophthalmologist collaboration are essential for timely detection and management. Future research should quantify the relative contribution of hypoalbuminemia, hypertension, and immunosuppression to ocular permeability changes, and evaluate preventive strategies tailored to high-risk CKD subgroups.

## 1. Introduction

End-stage chronic kidney disease (ESKD) is the final stage of chronic kidney disease (CKD), characterized by a glomerular filtration rate (GFR) less than 15 mL/min/1.73 m^2^, necessitating renal replacement therapy in the form of dialysis or kidney transplantation to sustain life [1]. The systemic consequences of ESKD are profound and multifactorial, including severe fluid overload, electrolyte imbalances, anemia, and cardiovascular disease [2]. Ocular alterations associated with chronic kidney disease (CKD) are numerous and well-documented in the literature: In the anterior segment, calcium deposition secondary to hyperparathyroidism and hypercalcemia may involve the conjunctiva and cornea, leading to chronic inflammation, pinguecula, and band-shaped keratopathy. Patients on dialysis often experience ocular surface dryness and discomfort due to reduced tear secretion and altered blink reflex. Cataract, particularly posterior subcapsular cataract, is more frequent and may develop rapidly [3].

The posterior segment complications include hypertensive retinopathy, diabetic retinopathy, central serous chorioretinopathy (CSCR), and serous retinal detachments (SRD). For the purposes of this literature review, we have chosen to focus specifically on changes affecting the choroidal and outer retina microcirculation and consequent permeability changes. These alterations often lead to the accumulation of serous fluid at the interface between the retinal pigment epithelium (RPE) and the neurosensory retina. As a result, patients affected by CKD may develop central serous chorioretinopathy (CSCR) and serous (transudative) retinal detachments (SRD) [4].

This review aims to explore the underlying mechanisms, the incidence, and epidemiologic factors linking the microvascular dysfunction in CKD to these specific ocular manifestations, focusing on choroidal vascular hyperpermeability, impaired RPE function, and the disruption of the outer blood-retinal barrier. We conducted an overview of the existing literature, also including several clinically relevant case reports, with the aim of synthesizing significant information regarding the ocular alterations described to date.

We seek to clarify how systemic renal impairment can manifest in the posterior segment of the eye, potentially guiding early detection of these manifestations, particularly among non-ophthalmologist clinicians, emphasizing the importance of early detection and interdisciplinary collaboration to improve visual outcomes in this patient population.

How do choroidal vascular hyperpermeability and dysfunction of the outer blood-retinal barrier contribute to the development of CSCR and serous retinal detachment in patients with end-stage chronic kidney disease? Can early multimodal imaging (OCT, OCT-A, fluorescein/ICG angiography) help detect subclinical choroidal and outer retinal changes in CKD patients, and could this guide preventive ophthalmologic surveillance? We hypothesize that the increased incidence of CSCR and SRD in transplant patients is attributable to multiple factors: partly due to reduced renal function, partly due to corticosteroid and immunosuppressive therapy, partly due to hypertension, and finally, to the heightened adrenergic response associated with the traumatic stress of surgery and the postoperative period [5]. This knowledge gap secondary to the limited understanding of choroidal vascular hyperpermeability and outer blood–retinal barrier dysfunction in patients with ESKD limits the clinician’s ability to identify at-risk patients and to implement appropriate surveillance strategies. Therefore, this review aims to synthesize current evidence and highlight the need for interdisciplinary research that clarifies these mechanisms and supports earlier ophthalmologic intervention in this vulnerable population.

## 2. Materials and Methods

We conducted an extensive review of the relevant articles published up to March 2025 to gather significant evidence regarding posterior segment complications in patients with chronic kidney disease (CKD). The data included in this review were collected from multiple sources, including the Medline database, the reference lists of selected articles, and authoritative ophthalmology and nephrology textbooks. This multi-source approach allowed for a comprehensive understanding of the topic and its conclusions.

Studies published in English were included if they reported clinical or experimental data on choroidal, retinal, or outer blood–retinal barrier alterations in patients with chronic kidney disease (CKD) or end-stage kidney disease (ESKD), including those undergoing hemodialysis, peritoneal dialysis, or renal transplantation. Eligible studies provided quantitative or qualitative assessments of choroidal or retinal thickness by OCT/EDI-OCT, angiographic findings, or data on the incidence of serous retinal detachment (SRD) and central serous chorioretinopathy (CSCR) in CKD populations.

Exclusion criteria included studies unrelated to the ocular effects of CKD, articles lacking clinical or imaging data on retinal or choroidal structure or function, narrative reviews without a defined methodology, non–full-text publications, and studies that did not specify renal status or type of renal replacement therapy. Reports in which ocular findings were primarily attributable to inflammatory, infectious, or neoplastic causes unrelated to renal dysfunction were also excluded.

The preliminary search of the literature was conducted to identify studies reporting keywords relevant to our research. In total, 144 articles were analyzed, and from these, we compiled three summary tables to provide a clear overview of the findings. The reviewed literature included both clinical studies and case reports that highlighted the spectrum of the posterior segment complications in CKD patients. A substantial portion of the information used in this paper was sourced from publications related to CSCR and serous retinal detachments, where details concerning chronic kidney disease were described.

Particular attention was given to studies that established a pathophysiological link between renal dysfunction and ocular manifestations, especially those implicating fluid dysregulation, choroidal and retinal circulation abnormalities, and the systemic implications.

### Method of Literature Search

- *Sources consulted*: MEDLINE database, PubMed, Scopus, and Google Scholar; reference lists of selected articles; and authoritative ophthalmology and nephrology textbooks.
- *Timeframe:* The search covered publications from 1980 to March 2025.
- *Search strategy*: Preliminary search conducted to identify studies using keywords relevant to posterior segment complications in chronic kidney disease (CKD).
- *Articles analyzed*: 144 studies in total, including clinical trials, observational cohorts, and case reports.
- *Inclusion focus*:
  ○ Studies establishing pathophysiological links between renal dysfunction and ocular manifestations.
  ○ Particular emphasis on fluid dysregulation, choroidal and retinal circulation abnormalities, and systemic factors.
- *Data abstracted*:
  ○ Choroidal thickness changes.
  ○ Incidence of central serous chorioretinopathy (CSCR) and serous retinal detachments (SRD) in dialysis and transplant populations.
  ○ Systemic variables: oncotic pressure, hypertension, corticosteroid exposure, and retinal pigment epithelium (RPE) pump function.

## 3. Pathophysiological Bases

A serous retinal detachment (SRD) is a fluid accumulation beneath the neurosensory retina without a retinal tear or tractional forces exerted on the retinal surface. While a leak from the choroid through the RPE must occur for fluid to enter the subretinal space, the key pathological feature is not just fluid entry but its persistence. Normally, the RPE actively transports fluid from the subretinal space to the choroid, making the system highly efficient—removing even protein-rich fluid within hours. However, if the RPE’s metabolic function is impaired, while its structural barrier remains intact, this active transport is disrupted. As a result, even small leaks can lead to persistent fluid accumulation [6].

This dysfunction may result from vascular abnormalities, inflammation, hypotony, or other metabolic insults that raise choroidal pressure or reduce intraocular pressure, encouraging fluid movement into the subretinal space. In diseases like central serous chorioretinopathy (CSCR), the leak is often focal, but the detachment is widespread, indicating diffuse RPE dysfunction is the main driver. This dysfunction reduces retinal adhesiveness and the ability of the RPE to clear fluid, allowing subretinal accumulation despite a small leak [7].

The blood-retinal barrier (BRB) is composed of two parts: the inner BRB (iBRB) and the outer BRB (oBRB). It plays a crucial role in regulating the exchange of fluids and molecules between the blood and the retina, protecting retinal tissue from harmful substances. The iBRB is formed by tight junctions between endothelial cells of the retinal vasculature, supported by astrocytes, Müller cells, and pericytes, which modulate barrier integrity and function. The oBRB is established by tight junctions between retinal pigment epithelial (RPE) cells, which sit atop Bruch’s membrane and control nutrient transport and waste removal between the choroid and photoreceptors. Both barriers maintain retinal homeostasis by limiting paracellular diffusion and enabling active transport. Disruption of the BRB—e.g., by matrix metalloproteinases—can impair its protective function. Cell polarity and tight junction proteins like occludin and ZO-1 are key to maintaining this selective permeability [8].

## 4. Systemic Factors Contributing to Serous Retinal Detachment in CKD

The homeostasis of retinal fluid and metabolites exchanges is ensured by a proper functioning inner and outer blood-retinal barrier, the latter being of relevance in our review. The integrity of the Outer BRB is essential in maintaining proper adhesion between the RPE and both the overlying neurosensory retina and the underlying Bruch’s membrane. The disruption of the Outer BRB, as observed in several pathological conditions, can potentially lead to fluid accumulation in the subretinal space.

In CKD, including Hemodialysis and renal transplant patients, the pathophysiological mechanisms underlying disruption of the oBRB and consequent subretinal fluid accumulation—manifesting clinically as serous retinal detachment or central serous chorioretinopathy (CSCR)—to date are not yet fully understood. Multiple studies suggest a central role of the imbalance among the 3 main factors:

  I. Intraocular pressure,
 II. Hydrostatic pressure within the choriocapillaris vascular lumen, and
III. The plasmatic oncotic pressure.

Among these, plasma oncotic pressure is markedly reduced in ESKD due to hypoalbuminemia and hyperalbuminuria. Under physiological conditions, the oncotic pressure of plasma in the choriocapillaris vascular lumen exceeds that of the vitreous, promoting passive fluid movement from the vitreous cavity into the vascular lumen—even in the presence of partial RPE dysfunction [9].

Systemic hypoalbuminemia–commonly observed in this population–may lead to a reduction in oncotic pressure within the choriocapillaris. This could favor transudation of fluid from the vascular lumen of the CC into the surrounding interstitial space. In more advanced cases, this can result in the accumulation of subretinal fluid and subsequent serous detachment of the neurosensory retina from the underlying RPE [10]. Supporting this hypothesis is the clinical observation that similar serous retinal detachments have been described in case reports of other conditions associated with chronic protein loss and reduced oncotic pressure, such as aggressive diuretic use or protein-losing enteropathies.

An angiographic study conducted by J. Donald and M. Gass [11] investigated fluorescein angiography (FA) features in patients undergoing hemodialysis who developed bullous retinal detachment. The study described a rare clinical presentation consisting of simultaneous, bilateral bullous retinal detachment characterized by multiple retinal pigment epithelium (RPE) detachments, each surrounded by gray, white subretinal exudates. Notably, these findings occurred in the absence of inflammatory signs or rhegmatogenous lesions [11].

The angiographic evidence, as detailed by the authors, supports the presence of focal alterations in choriocapillaris permeability. These changes appear sufficient to permit leakage of large molecules, such as fibrinogen, into the sub-RPE and subretinal spaces. Importantly, this phenomenon was observed in the absence of choroiditis or other intraocular inflammatory signs typically associated with increased vascular permeability, thereby broadening the differential diagnosis in patients presenting with non-inflammatory exudative retinal detachment.

These findings underscore the need for clinicians to consider ESKD-related systemic factors when evaluating atypical bilateral retinal detachments, especially in the absence of conventional inflammatory or rhegmatogenous etiologies [11].

To date, no certain correlation is available between subretinal transudation and eGFR and albuminemia levels. However, identifying a clear and direct relationship between these parameters and the risk of SRF accumulation would be of substantial clinical value for earlier identification of at-risk patients.

An additional consideration well documented in the literature and in major clinical guidelines is that patients with CKD are frequently affected by concomitant arterial hypertension. In some cases, hypertension presents as a comorbidity, while in others, it constitutes the primary factor responsible for the progressive decline in renal function over time [11]. In the studies analyzed, up to 89% of the selected sample population was found to have arterial hypertension, which is itself a contributing factor to the transudation of fluid into the subretinal space, increasing the risk of neuroretinal detachment, as observed in these patients [5]. Furthermore, it is relevant to note that the population of patients affected by CKD—particularly those undergoing kidney transplant or suffering from autoimmune-based glomerulonephritis—are routinely exposed to immunosuppressive therapies involving corticosteroids and cyclosporine, which are both agents significantly associated with the development or exacerbation of arterial hypertension [12]. The study by Lahme et al. (2023) [13] demonstrated that patients with end-stage renal disease (ESRD) undergoing hemodialysis exhibited reduced flow density (FD) in the superficial capillary plexus (SCP) and choriocapillaris (CC) on optical coherence tomography angiography (OCTA) compared to matched healthy controls, and that retinal thickness and volume were also significantly decreased; notably, deeper plexuses such as the deep capillary plexus (DCP) appeared relatively preserved. Direct data on peritoneal dialysis (PD) and ocular microvasculature are still scant; the inference is that alternative dialysis modalities may also influence ocular microvascular parameters [13].

## 5. Choroidal and Retinal Micro-Anatomy in Hemodialysis Patients

Retinal and choroidal microcirculatory alterations in ESKD contribute to posterior segment pathology. Several studies in the literature analyze the anatomical and functional changes in the retina and choroid in patients with end-stage CKD, both before and after hemodialysis. During hemodialysis, systemic and metabolic changes occur, including fluid loss, reduced plasma osmolarity and thus body mass, and increased oncotic pressure [14].

Animal studies have demonstrated the reduced autoregulatory capacity of choroidal vessels, which are therefore significantly affected by systemic hemodynamic and metabolic changes—factors that are common to all patients with ESKD—potentially leading to impaired perfusion of the outer retinal layers [15].

A parameter of interest among authors concerns the variation in choroidal thickness measured with EDI-OCT in the same patients before and after undergoing routine hemodialysis. In a study conducted by In Boem Chang et al. [16], a group of subjects with end-stage CKD were subjected to choroidal thickness measurements during hemodialysis. Measurement points were defined in two areas: central choroidal thickness, measured at the fovea and 1.5 mm temporally from the fovea; and peripheral choroidal thickness, measured superiorly, nasally, and inferiorly to the optic disc.

To minimize the influence of pre-existing diabetic retinopathy, patients were divided into diabetic and non-diabetic groups, and the pre- and post-hemodialysis differences were calculated for each group. The study demonstrated a statistically significant variation in choroidal thickness at all defined points for both groups of patients, with a mean reduction of 22.8 microns in central choroidal thickness after hemodialysis in the diabetic group, and a mean to the same point of 15.1 microns in the non-diabetic group. Regarding the measurement of the peripheral non-central points, the mean change of the choroidal thickness recorded was 17.4 microns at the nasal point to the optic disc in the diabetic group, and 12.3 microns at the corresponding point in non-diabetic patients [16], demonstrating that the systemic and metabolic changes before and after hemodialysis also have an effect on choroidal circulation [17].

Other studies have confirmed these clinical findings, highlighting the difference in choroidal thickness pre- and post-hemodialysis, particularly in patients with diabetes mellitus compared to non-diabetic patients [18]. These findings support the theory that the transient reduction in plasma colloid osmotic pressure could lead to fluid accumulation in the choroidal interstitium [18]. Hemodialysis, by restoring a physiological colloid-osmotic pressure [19], results in a rapid decrease in choroidal thickness due to a reduction in interstitial fluid volume, facilitated by the high permeability of the highly fenestrated choroidal vasculature [20].

An additional proposed mechanism is that the choroidal vascular smooth muscle constriction, caused by sympathetic activation triggered by blood volume depletion, could lead to a decrease in choroidal thickness [21].

Some authors have also reported a reduction in chronic macular edema following the initiation of hemodialysis. This effect has been hypothesized to result from changes in the concentration of nitric oxide synthase inhibitors, which decrease after dialysis, thereby enhancing systemic nitric oxide (NO) bioavailability. The increased availability of NO may exert vasodilatory effects on the choroidal vasculature, potentially contributing to the resolution of macular edema [22,23].

With respect to the hypothesis that ocular hemodynamic changes induced by hemodialysis may underlie the angiographic findings previously described, several studies have investigated the hemodynamics of the ocular and facial circulation. These studies have evaluated parameters such as blood flow velocity and resistance index in the ophthalmic artery, ciliary arteries, and central retinal artery. The findings consistently demonstrate a significant decrease in blood flow velocity following hemodialysis when compared to pre-dialysis values [24].

These observations suggest that the post-dialysis reduction in hydrostatic pressure may lower the driving force responsible for interstitial fluid outflow. Consequently, this reduction may facilitate the reabsorption of subretinal fluid, offering a potential explanation for the spontaneous resolution of non-inflammatory subretinal fluids observed in some patients undergoing hemodialysis [25].

The link between hemodialysis and choroidal thickness is widely described in the literature [25]. However, there is uncertainty regarding a statistically significant correlation between hemodialysis and Retinal thickness (RT). Several studies have attempted to validate a correlation between retinal thickness—calculated using a macular scan according to the ETDRS grid—and systemic changes before and after hemodialysis in patients with end-stage CKD, but the results conclude that although variations are present, they are minimal and not statistically significant, as no significant changes are observed in any of the ETDRS grid subfields after hemodialysis [14].

We hypothesize that the reason why retinal thickness is not influenced by systemic fluid and electrolyte balance—and therefore does not vary following hemodialysis, unlike choroidal thickness—may be attributed to the significantly greater autoregulatory capacity of the retinal vascular circulation and the presence of the inner blood-retinal barrier [26]. This contrasts with what has been previously described regarding the choroidal vasculature, which possesses a lower autoregulatory capacity in response to systemic changes, as it is highly fenestrated and permeable. [27].

However, most studies present the limitation of correlating the hemodialysis process solely with anatomical data, such as variations in retinal and choroidal thickness, without considering functional data, such as changes in perfused vessel density of the choroid and retina. In this regard, the study conducted by Yong Un Shin is particularly significant: all enrolled patients underwent SS-OCT-A imaging to assess the vascular perfusion density of both the choriocapillaris and the intraretinal capillary plexuses before and after hemodialysis, generating color-coded perfusion density maps for both the choroid and the retina.

The study concluded that there is no statistically significant correlation between the variation in perfused vessel density in the choriocapillaris and the variation in choroidal thickness in any of the subfields of the ETDRS grid. Furthermore, it also found no statistically significant correlation between changes in perfused vessel density in either the superficial or deep capillary plexus and variations in retinal thickness across any of the ETDRS grid subfields, concluding that the fluid and electrolyte imbalances observed in patients with end-stage CKD undergoing hemodialysis are not responsible for changes in choroidal or retinal perfused vessel density [28].

Finally, it is important to consider that the retinal and choroidal changes described following hemodialysis may be influenced by other confounding factors, such as significant body weight loss, reduction in systolic and diastolic blood pressure, and decreased ocular perfusion pressure—factors that may account for the observed anatomical variations [23,28].

With the aim of further enhancing the completeness of the present review, we have compiled and presented in Table 1 the values of choroidal thickness, along with their documented variations in relation to the hemodialysis procedures to which the patients were subjected. These data have been gathered from the studies available in the current ophthalmologic and nephrology literature to summarize the choroidal changes.

**Table 1 Summary**

A consistent reduction in choroidal thickness following hemodialysis was reported across most studies. Prospective before–after investigations, including those by **Shoshtari et al. (2021) [29]**, **Sun et al. (2019) [30]**, **Ulas et al. (2013) [17]**, **Shin et al. (2018) [28]**, **Yang et al. (2013) [31]**, **Zegrari et al. (2023) [32]**, and others, demonstrated significant decreases in subfoveal and regional choroidal thickness, typically ranging between **−10 µm and −25 µm**, although some studies reported larger reductions (e.g., **Chen et al. (2018) [39]**, **−35 µm**) or minor/non-significant changes (**Kang et al. (2017) [40]**). The reduction was generally observed across nasal, temporal, and subfoveal measurements, regardless of baseline thickness or patient subgroup. A few exceptions were noted: **Jung et al. (2014) [38]** observed a mild post-dialysis increase (+11 µm), while **Coppolino et al. (2022) [36]** found that patients experiencing intradialytic hypotension exhibited a greater decrease compared to non-hypotensive individuals. Several studies also assessed the influence of diabetes, such as **Ishibazawa et al. (2015) [18]** and **Nakano et al. (2020) [42]**, showing that diabetic patients tended to experience greater thinning than their non-diabetic counterparts. Overall, these findings indicate that hemodialysis induces a transient but measurable reduction in choroidal thickness—likely reflecting fluid shifts and osmotic changes—while retinal thickness alterations were less pronounced and inconsistently reported.

## 6. Retinal Detachment and CSCR in End-Stage CKD and Hemodialysis

The association between serous retinal detachment (SRD) and chronic kidney disease (CKD), particularly in end-stage CKD, is well established in the literature and recognized by clinicians. One of the largest cohort studies examining the link between SRD and CKD, conducted by Yuh-Shin Chang et al. [43], included over 94,000 patients from the Taiwan region. The study demonstrated a significantly higher odds ratio for developing SRD among patients undergoing hemodialysis due to end-stage CKD compared to control subjects. In this study, among the 94,024 patient selected, 27 patients of the hemodialysis group developed SRD (representing 0.03%), while only 11 cases were observed in the control group of patient not undergoing hemodialysis (0.01%) and the incidence rate was 0.61 per 10,000 person-years (PY) in the ESKD group versus 0.18 per 10,000 PY in controls, yielding a statistically significant incidence rate ratio (IRR) of 3.39 (95% CI: *p* < 0.001)

A significant finding highlighted by the authors is the higher incidence rate of serous retinal detachment (SRD) and central serous chorioretinopathy (CSCR) among patients aged between 50 and 64 years, followed by those aged >65 years and >50 years. Additionally, a greater incidence of SRD was observed in male patients compared to females, both within the hemodialysis population and in the control group of non-dialyzed patients [43].

The same authors, similarly to what was done with the SRD, compared the risk of CSCR incidence in patients with ESKD undergoing dialysis to a control group, identifying a statistically significant difference in incidence between the two groups: During the follow-up period, the incidence of CSCR in the ESKD group was 5.05 per 10,000 person-years (PY), compared to 3.34 per 10,000 PY in controls, with an incidence rate ratio (IRR) of 1.51 (*p* < 0.0001).

A sex-based analysis also showed significantly increased CSCR incidence in male (IRR = 1.43, *p* = 0.0098) and female ESKD patients (IRR = 1.60, *p* = 0.0015) compared to their respective controls.

Another relevant observation is the higher incidence rate of SRD in patients undergoing peritoneal dialysis (PD) compared to those receiving conventional hemodialysis. The authors suggested that the increased incidence associated with PD may be attributed to chronic inflammation resulting from the peritoneal glucose load, as well as to chronic vascular injury and consequent endothelial hyperpermeability mediated by advanced glycation end products (AGEs) generated through persistent peritoneal glucose exposure [43].

In their conclusion, the authors state that both hemodialysis and peritoneal dialysis represent independent risk factors for the development of serous retinal detachment in patients with ESKD, independently of other comorbidities. Therefore, they recommend that nephrologists maintain close collaboration with ophthalmologists whenever a decrease in visual acuity is observed in dialysis patients, given the potential involvement of the posterior segment.

For instance, a study conducted on a large sample of 1529 individuals from the northern Chinese population affected by CKD, defined as reduced eGFR, showed no correlation between kidney failure and any major retinal diseases [44] except for retinal vein occlusion, standing in contrast to the conclusions previously discussed. Such discrepancies highlight the need for further research to clarify the relationship between renal dysfunction and ocular health, considering possible differences in population characteristics, study design, and definitions of disease outcomes.

## 7. Renal Transplant

Renal transplantation is a common indication for long-term corticosteroid therapy as part of immunosuppressive regimens, and is often responsible for CSCR and SRD in the renal transplanted patients, considering the increased risk of CSCR and SRD in CCS users [44].

Cases of severe, often bilateral, visual loss occurring days or weeks after kidney transplantation have been documented in the literature. As reported by Scorolli et al., visual function following renal transplantation should not be underestimated. While minor visual changes may be attributed to transient electrolyte imbalances or fluctuations in the refractive index of the eye’s aqueous media, more serious underlying causes should also be considered—such as serous bullous retinal detachments.

In this context, the Amsler grid proves to be a valuable and practical tool, even for bedridden patients. It is inexpensive, easy to administer in detecting early visual disturbances. These may be caused by subretinal fluid accumulation, which can distort the foveal contour and alter central vision.

In the case series reported by Scorolli, all four patients experienced normalization of ocular findings within a few weeks after renal function returned to normal levels. This was accompanied by recovery of visual acuity and a return to normal Amsler grid testing, although some residual pigmentary changes were observed during fundus examination [45].

In these and similar cases, the subretinal fluid accumulation is not solely due to electrolyte imbalances. One must also consider that these patients are exposed to significant stress related to advanced disease, the transplant procedure itself, and hospitalization, in addition to the corticosteroid treatment for immunosuppression, which are well known to increase the risk of developing central serous chorioretinopathy (CSCR) and serous detachments of the neurosensory retina [46].

A significant retrospective study conducted between 1979 and 2009 on 451 Korean patients with end-stage CKD who underwent kidney transplantation aimed to clarify the actual correlation between kidney transplantation and the occurrence of serous subretinal fluid accumulation manifestations, such as CSCR. The study reports that 39 eyes from 28 patients developed CSCR following kidney transplantation, with a prevalence rate of 6%. The mean age of affected patients was 46.4 years, with a higher prevalence in males (64% of the total), and a wide time range between the transplantation and the onset of CSCR, spanning from 2 months to 20 years, thereby demonstrating a higher CSCR prevalence in this cohort [5].

However, it is important to consider that all the patients included in the study were under continuous corticosteroid therapy, with a daily dosage ranging between 5 and 10 mg, in combination with at least one other immunosuppressive agent. This represents a major confounding factor, as numerous studies in the literature have associated CSCR and choroidal disorders with prolonged corticosteroid use, showing significantly higher prevalence in patients using them for various conditions [47,48,49,50].

On the other hand, supporting the correlation between kidney transplantation and CSCR, some studies in the literature have reported a higher CSCR prevalence after kidney versus other solid organ transplants. This suggests corticosteroids alone may not fully explain the risk, but that kidney transplantation itself may play a role, especially considering that patients undergoing other solid organ transplants are also exposed to prolonged immunosuppressive therapies, including corticosteroids and other agents [51,52].

A relevant retrospective study conducted by Fawzi et al. analyzed the characteristics of retinal manifestations in patients following solid organ transplantation, specifically describing the clinical findings in those who developed central serous chorioretinopathy (CSCR) and bullous retinal detachment (RD). The study included 30 eyes from 15 patients who had undergone solid organ transplantation and subsequently developed CSCR or bullous RD months to years post-transplantation. Kidney transplantation showed the strongest association with these manifestations, accounting for 87% of cases, followed by liver (6.5%) and heart transplants (6.5%). The time interval between transplantation and the onset of visual symptoms was highly variable, ranging from immediately after surgery to up to 7 years later.

The authors also identified four distinct patterns of CSCR within the analyzed cohort, namely geographic retinal pigment epitheliopathy, focal CSCR, multifocal CSCR, and CSCR with bullous retinal detachment, all observed across the included cases. Another significant finding of this study was the presence of bilateral retinal changes in 83.3% of the patients.

Nevertheless, it is important to acknowledge the evident limitations of this study, primarily the small sample size, which precludes the determination of a true prevalence of CSCR following solid organ transplantation. Moreover, confounding factors—such as high-dose immunosuppressive therapy with agents like cyclosporine and prednisone, known for their vasogenic effects on the choroid—must be considered, as they inherently increase the risk of developing CSCR in transplanted patients [51].

Another factor to consider is that in the immediate postoperative period following kidney transplantation, there is an increased prevalence of arterial hypertension, which has been linked to a higher risk of serous retinal detachment and CSCR. This is due both to the significant corticosteroid therapy—because of its mineralocorticoid effect—and to the initial hypo-uria and reduced renal filtration capacity [53].

Since renal transplantation is typically reserved for life-threatening conditions, postoperative visual disturbances may be overlooked or underreported due to their perceived insignificance or transient nature. Furthermore, only patients presenting with symptomatic macular involvement are likely to come to clinical attention. Serous retinal detachments occurring outside the central macula, particularly in the posterior pole beyond the vascular arcades or in the peripheral retina, are often asymptomatic and therefore frequently go undetected or misinterpreted.

## 8. Conclusions

Changes in choroidal thickness and perfusion, though not always matched by functional alterations, point toward a complex interplay between systemic and ocular vascular regulation in ESKD.

Large-scale cohort studies, such as the one conducted by Yuh-Shin Chang et al., have demonstrated an increased incidence of SRD and central serous chorioretinopathy (CSCR) in patients undergoing hemodialysis compared to non-dialyzed controls. These retinal pathologies exhibit higher prevalence among middle-aged to elderly populations and show a predilection for male patients. However, the magnitude of this association varies between studies, likely reflecting differences in dialysis modality, fluid management, and systemic comorbidities. Peritoneal dialysis may confer a greater risk for SRD, possibly due to chronic inflammation and endothelial dysfunction induced by prolonged exposure to glucose-containing dialysis fluids.

Renal transplantation, while lifesaving, introduces additional complexities regarding retinal health. The widespread use of corticosteroids and other immunosuppressive agents as part of post-transplant therapy significantly elevates the risk of developing CSCR and serous retinal detachments. Visual impairment in post-transplant patients may also result from a combination of electrolyte imbalances, systemic hypertension, surgical stress, and immunosuppressive medication side effects. Notably, retinal complications may present months or even years after transplantation, underscoring the need for long-term ophthalmologic surveillance.

Some discrepancies exist in the literature, with certain studies failing to establish a clear correlation between renal dysfunction and retinal diseases, highlighting the influence of population heterogeneity, study design, and diagnostic criteria.

Overall, inconsistencies across the literature underscore several key uncertainties:

- The precise mechanistic link between choroidal perfusion changes and SRD/CSCR development remains unresolved.
- Population heterogeneity, study design, and OCT/OCTA protocols contribute to variability in reported outcomes.
- The relative contributions of hypoalbuminemia, blood pressure variability, and immunosuppressive therapy to outer blood-retinal barrier dysfunction are poorly quantified.

Observational studies report variable findings highlighting the influence of methodological differences, timing of measurements, and population heterogeneity. Furthermore, serous retinal detachments occurring outside the central macula often remain asymptomatic and may be underdiagnosed.

It would be recommended that nephrologists and ophthalmologists collaborate closely to monitor visual function in patients with advanced CKD, those undergoing dialysis, and renal transplant recipients. Early detection tools, such as the Amsler grid, are valuable for identifying subtle visual disturbances. A comprehensive understanding of the multifactorial pathophysiology will enhance patient care and potentially mitigate vision-threatening complications in this vulnerable population.

To conclude, the aim of the authors is to raise awareness among both ophthalmologists and non-ophthalmic healthcare professionals about the potential retinal and choroidal complications associated with chronic kidney disease and renal transplant, while also attempting to define their underlying pathogenic mechanisms; however, a definitive mechanism remains unidentified and requires further investigation.

We emphasize the importance of the ophthalmologist to carefully distinguish whether the ocular findings represent a primary ocular pathology or are a manifestation of an underlying systemic disease to avoid mistaking systemic causes for primary ocular disease. This distinction holds significant clinical implications, as it directly influences therapeutic decision-making and prognosis. In cases where SRDs are secondary to systemic conditions, particularly in the case of autoimmune disorders and renal dysfunction, the detachment typically responds favorably to systemic management with corticosteroids and immunosuppressive agents. Conversely, if the SRD stems from a primary ocular disorder, such as central serous chorioretinopathy (CSR), the use of systemic corticosteroids may exacerbate the condition.

This highlights the importance of a thorough systemic evaluation and a high index of suspicion, especially in patients presenting in non-ophthalmologic settings where visual symptoms might be overlooked or misinterpreted. Prompt identification of the underlying etiology not only guides appropriate treatment but also prevents iatrogenic worsening of the ocular pathology. Multidisciplinary collaboration between ophthalmologists and other specialists is therefore essential in optimizing outcomes for patients presenting with exudative retinal detachments.

Clinical recommendations:

- Ophthalmologists must distinguish primary ocular pathology from systemic-induced retinal changes to guide therapy appropriately.
- Multidisciplinary collaboration between nephrologists and ophthalmologists is essential for early detection and management.
- Simple screening tools such as OCT imaging and Amsler grid self-testing can facilitate timely identification of subretinal fluid and subtle RPE dysfunction.

Early recognition of ocular clinical findings and a thorough general diagnostic work-up is essential in cases of suspected systemic involvement, particularly in non-ophthalmologic settings, where visual symptoms may be underappreciated or misattributed.

## 9. Final Recommendations

Based on the current evidence, several recommendations can be made for clinical practice and future research. First, nephrologists and ophthalmologists should establish systematic collaboration to ensure early recognition of choroidal and retinal alterations in patients with advanced CKD, those on chronic dialysis, and post-transplant recipients. Simple tools such as Amsler grid self-testing and periodic OCT imaging may allow timely detection of subretinal fluid and subtle RPE dysfunction. Second, given the consistent evidence of reduced choroidal thickness following hemodialysis, future studies should clarify whether longitudinal OCT/OCTA monitoring can predict which patients are at highest risk for CSCR or SRD. Third, in the transplant setting, efforts should be directed toward minimizing corticosteroid exposure and controlling hypertension, while simultaneously monitoring ocular status. Finally, large prospective, multiethnic cohort studies are urgently needed to disentangle the contributions of hypoalbuminemia, hydrostatic fluctuations, and immunosuppressive therapy to outer BRB dysfunction. Such strategies may ultimately improve both visual prognosis and quality of life in this vulnerable patient population. The evidence synthesized in this review highlights the need for a more systematic evaluation of outer blood–retinal barrier dysfunction in patients with chronic kidney disease, particularly in those undergoing dialysis or receiving renal transplantation. Future research should prioritize longitudinal, multimodal imaging studies to quantify dynamic changes in choroidal structure and RPE function in relation to dialysis cycles, transplant phases, and immunosuppressive regimens. Moreover, large-scale, multiethnic cohorts are required to clarify the relative contributions of hypoalbuminemia, blood pressure variability, and corticosteroid exposure to the risk of CSCR and SRD. From a translational perspective, standardized protocols for OCT and OCTA monitoring in nephrology populations could provide valuable surrogate endpoints. Ultimately, advancing this field will require an integrative approach that bridges nephrology, ophthalmology, and vascular biology, with the goal of reducing vision-threatening complications in patients with advanced renal disease.

## Figures and Tables

**Table 1:** Effects of Hemodialysis on Choroidal Thickness Measurements.

	Study	Author (Year)	Choroidal Thickness Before HD	Choroidal Thickness After HD	Difference	Notes
**1**	***The impact of hemodialysis on retinal and choroidal thickness in patients with chronic renal failure***	**Shoshtari et al. (2021)** [29]	1000 µm Nasal: 275.51 ± 2.3 1000 µm Temporal: 291.47 ± 2.2	1000 µm Nasal: 259.50 ± 6.9 1000 µm Temporal: 271.52 ± 8.1	1000 µm Nasal: −16.01 µm 1000 µm Temporal: −19.95 µm	Prospective–Before-After
**2**	***The effect of hemodialysis on ocular changes in patients with end-stage renal disease***	**Sun et al. (2019)** [30]	Subfoveal: 254.29 ± 69.36	Subfoveal: 235.54 ± 59.90	Subfoveal: −18.75	Cross sectional
**3**	***Evaluation of choroidal and retinal thickness in non-diabetic haemodialysis patients***	**Ulas et al. (2013)** [17]	Nasal (1500 µm): 182.14 ± 68.88 Temporal (1500 µm): 212.43 ± 70.50 Subfoveal: 232.81 ± 71.92	Nasal (1500 µm): 165.19 ± 66.73 Temporal (1500 µm): 195.38 ± 66.48 Subfoveal: 210.90 ± 65.53	Nasal (1500 µm): −16.95 Temporal (1500 µm): −17.05 Subfoveal: −21.91	Prospective–Before-After
**4**	***Optical coherence tomography angiography of retina and choroid after haemodialysis***	**Shin et al. (2018)** [28]	Center (C): 214.8 ± 80.9 Outer Superior (OS): 219.4 ± 70.4 Outer Nasal (ON): 182.3 ± 58.6 Outer Inferior (OI): 194.3 ± 68.8 Outer Temporal (OT): 207.6 ± 60.7 Inner Superior (IS): 224.3 ± 76.1 Inner Nasal (IN): 214.5 ± 75.9 Inner Inferior (II): 214.4 ± 74.1 Inner Temporal (IT): 214.4 ± 68.7 Global Mean (TCT): 209.6 ± 64.6	Center (C): 199.1 ± 83.2 Outer Superior (OS): 208.0 ± 67.6 Outer Nasal (ON): 164.6 ± 56.3 Outer Inferior (OI): 179.9 ± 64.4 Outer Temporal (OT): 194.4 ± 61.3 Inner Superior (IS): 210.9 ± 76.6 Inner Nasal (IN): 196.5 ± 75.7 Inner Inferior (II): 199.5 ± 72.3 Inner Temporal (IT): 203.4 ± 70.9 Global Mean (TCT): 195.2 ± 64.1	Center (C): −15.7 Outer Superior (OS): −11.4 Outer Nasal (ON): −17.7 Outer Inferior (OI): −14.4 Outer Temporal (OT): −13.2 Inner Superior (IS): −13.4 Inner Nasal (IN): −18.0 Inner Inferior (II): −14.9 Inner Temporal (IT): −11.0 Global Mean (TCT): −14.4	Prospective–Before-After
**5**	***Changes in choroidal thickness, intraocular pressure, and other optical coherence tomographic parameters after haemodialysis***	**Yang et al. (2013)** [31]	Average Subfoveal: 233.1 ± 77.5	Average Subfoveal: 219.1 ± 76.8	Average Subfoveal: −14.0	Prospective–Before-After
**6**	***Optical coherence tomography angiography analysis of changes in the retina and the choroid after hemodialysis for end-stage kidney disease***	**Zegrari et al. (2023)** [32]	Subfoveal: 234.3 ± 56.1	Subfoveal: 211.9 ± 60.8	Subfoveal: −22.4	Prospective–Before-After
**7**	***Effect of Hemodialysis on Eye Coats, Axial Length, and Ocular Perfusion Pressure in Patients with Chronic Renal Failure***	**Wang et al. (2018)** [33]	Nasal (1500 µm): 197.84 ± 95.43 Temporal (1500 µm): 233.37 ± 84.88 Subfoveal: 267.97 ± 99.17	Nasal (1500 µm): 186.29 ± 91.44 Temporal (1500 µm): 219.66 ± 83.04 Subfoveal: 255.87 ± 95.59	Nasal (1500 µm): −11.55 Temporal (1500 µm): −13.71 Subfoveal: −12.10	Prospective–Before-After
**8**	***Changes in retina and choroid after haemodialysis assessed using optical coherence tomography angiography***	**Zhang et al. (2018)** [34]	Subfoveal: 240.3 ± 91.7	Subfoveal: 228.4 ± 82.3	Subfoveal: −11.9	Prospective–Before-After
**9**	***Changes In Choroidal Thickness In And Outside The Macula After Hemodialysis In Patients With End-Stage Renal Disease***	**Chang et al. (2017)** [16]	Foveal center (0 µm): 233.6 ± 45.2 Temporal 1500 µm from fovea: 211.1 ± 42.7 Superior 3500 µm from optic disc: 207.3 ± 15.3 Inferior 3500 µm from optic disc: 173.2 ± 31.5 Nasal 3500 µm from optic disc: 181.3 ± 48.1	Foveal center (0 µm): 214.2 ± 43.8 Temporal 1500 µm from fovea: 196.1 ± 41.0 Superior 3500 µm from optic disc: 193.4 ± 15.7 Inferior 3500 µm from optic disc: 157.9 ± 29.1 Nasal 3500 µm from optic disc: 166.1 ± 46.9	Foveal center (0 µm): −19.4 Temporal 1500 µm from fovea: −15.0 Superior 3500 µm from optic disc: −13.9 Inferior 3500 µm from optic disc: −15.3 Nasal 3500 µm from optic disc: −15.2	Prospective–Before-After
**10**	***The Acute Effect of Hemodialysis on Choroidal Thickness***	**Celikay et al. (2015)** [35]	Subfoveal: 254.59 ± 84.66 Temporal (1500 µm): 237.83 ± 83.97 Temporal (3000 µm): 209.29 ± 64.17 Nasal (1500 µm): 197.85 ± 73.92 Nasal (3000 µm): 131.88 ± 61.86	Subfoveal: 229.34 ± 77.79 Temporal (1500 µm): 214.90 ± 76.62 Temporal (3000 µm): 194.90 ± 64.42 Nasal (1500 µm): 183.32 ± 67.10 Nasal (3000 µm): 115.61 ± 56.76	Subfoveal: −25.24 Temporal (1500 µm): −22.93 Temporal (3000 µm): −14.40 Nasal (1500 µm): −14.54 Nasal (3000 µm): −16.27	Prospective–Before-After
**11**	***Acquisition of optical coherence tomography angiography metrics during hemodialysis procedures: A pilot study***	**Coppolino et al. (2022)** [36]	Subfoveal (All): 193.83 ± 43.88 Subfoveal (Hypotensive): 195.90 ± 52.42 Subfoveal (Non-Hypotensive): 173.25 ± 33.04	Subfoveal (All): 181.82 ± 47.77 Subfoveal (Hypotensive): 170.22 ± 56.87 Subfoveal (Non-Hypotensive): 174.75 ± 45.18	All: −12.01 Hypotensive: −25.68 Non-Hypotensive: +1.50	Prospective–Before-After
**12**	***Are the effects of hemodialysis on ocular parameters similar during and after the session?***	**Elbay et al. (2017)** [37]	Subfoveal: 270.85 ± 73.82	Subfoveal: 258.44 ± 75.17	Subfoveal: −12.41	Prospective–Before-After
**13**	***Choroidal Thickness Before and After Hemodialysis in Diabetic and Non-Diabetic Patients***	**Ishibazawa et al. (2015)** [18]	DM (Subfoveal): 268 ± 75 NDM (Subfoveal): 233 ± 78	DM (Subfoveal): 234 ± 69 NDM (Subfoveal): 217 ± 72	DM (Subfoveal): −34 NDM (Subfoveal): −16	Diabetic (DM) Vs Non-Diabetic (NDM)
**14**	***Changes in subfoveal choroidal thickness and choroidal extravascular density by spectral domain optical coherence tomography after hemodialysis: A pilot study***	**Jung et al. (2014)** [38]	Subfoveal: 276.94 ± 58.73	Subfoveal: 288.29 ± 65.57	Subfoveal: +11.35	Prospective–Before-After
**15**	***Ocular changes during hemodialysis in patients with end-stage renal disease***	**Chen et al. (2018)** [39]	Subfoveal: 289.55 ± 11.385	254.134 ± 11.46	−35.42 µm	Prospective–Before-After
**16**	***Changes in Choroidal Thickness after Hemodialysis and the Influence of Diabetes***	**Kang et al. (2017)** [40]	Subfoveal (All): 311.8 ± 64.9 Subfoveal; Diabetic group (DM): 316.4 ± 32.0 Subfoveal; Non diabetic group (Non-DM): 316.1 ± 104.5	Subfoveal (All): 311.2 ± 65.1 Subfoveal (DM): 307.7 ± 45.7 Subfoveal (Non-DM): 315.5 ± 84.9	All: −0.6 DM: −8.7 Non-DM: −0.6	Prospective–Before-After
**17**	***Evaluation of choroidal and retinal thickness measurements in adult hemodialysis patients using spectral-domain optical coherence tomography***	**Kal et al. (2016)** [41]	Subfoveal: 182 (range 103–374)	Subfoveal: 161 (range 90–353)	Subfoveal: −21	Prospective–Before-After
**18**	***Choroid structure analysis following initiation of hemodialysis by using swept-source optical coherence tomography in patients with and without diabetes***	**Nakano et al. (2020)** [42]	Subfoveal DM: 301.7 ± 70.3 Subfoveal NDM: 281.8 ± 57.1	Subfoveal DM: 261.6 ± 77.3 Subfoveal NDM: 259.8 ± 68.3	DM: −40.1 NDM: −22.0	Prospective–Before-After

**Table 1**—Changes in choroidal thickness assessed through Optical Coherence Tomography (OCT) performed before and after hemodialysis.

## Data Availability

Not applicable, as no new data were created or analyzed in this study.

## References

[1] Acosta-Ochoa I., Bustamante-Munguira J., Mendiluce-Herrero A., Bustamante-Bustamante J., Coca-Rojo A. (2019). Impact on outcomes across KDIGO-2012 AKI criteria according to baseline renal function. J. Clin. Med..

[2] Go A.S., Chertow G.M., Fan D., McCulloch C.E., Hsu C. (2004). Chronic Kidney Disease and the Risks of Death, Cardiovascular Events, and Hospitalization. N. Engl. J. Med..

[3] Shanti Y., Hamayel H., Yasin A., Abu Shanab A., Hroub O., Hamdan Z., Shraim M. (2023). Epidemiology of Common Ocular Manifestations among Patients on Haemodialysis in West Bank, Palestine. Sultan Qaboos Univ. Med. J..

[4] Wong C.W., Wong T.Y., Cheng C.Y., Sabanayagam C. (2014). Kidney and eye diseases: Common risk factors, etiological mechanisms, and pathways. Kidney Int..

[5] Lee C.S., Kang E.C., Lee K.S., Byeon S.H., Koh H.J., Lee S.C. (2011). Central serous chorioretinopathy after renal transplantation. Retina.

[6] Fossataro F., Fossataro C., Abraham N., Fouad Y., Mrejen S., Tan A.C., Voichanski S., Sarraf D. (2024). Pathogenesis of Central Serous Chorioretinopathy and the Link Between Choroidal Hyperpermeability and Retinal Pigment Epithelium Pump Reversal. Am. J. Ophthalmol..

[7] Zarnegar A., Ong J., Matsyaraja T., Arora S., Chhablani J. (2023). Pathomechanisms in central serous chorioretinopathy: A recent update. Int. J. Retin. Vitr..

[8] Cunha-Vaz J., Bernardes R., Lobo C. (2011). Blood-retinal barrier. Eur. J. Ophthalmol..

[9] Ghazi N.G., Green W.R. (2002). Pathology and pathogenesis of retinal detachment. Eye.

[10] Henriques S., Lima A., Almeida J., Basto R., Roque J., Coutinho I., Prieto I. (2021). Bilateral retinal detachment—When the kidney meets the eye Descolamento de retina bilateral—Quando o rim afeta o olho. Rev. Bras. Oftalmol..

[11] Donald J., Gass M. (1992). Bullous retinal detachment and multiple retinal pigment epithelial detachments in patients receiving hemodialysis. Graefe’s Arch. Clin. Exp. Ophthalmol..

[12] Cunningham E.T., Alfred P.R., Irvine A.R. (1996). Central serous chorioretinopathy in patients with systemic lupus erythematosus. Ophthalmology.

[13] Lahme L., Storp J.J., Marchiori E., Esser E., Eter N., Mihailovic N., Alnawaiseh M. (2023). Evaluation of Ocular Perfusion in Patients with End-Stage Renal Disease Receiving Hemodialysis Using Optical Coherence Tomography Angiography. J. Clin. Med..

[14] Chelala E., Dirani A., Fadlallah A., Slim E., Abdelmassih Y., Fakhoury H., Baz P., Bejjani R. (2015). Effect of hemodialysis on visual acuity, intraocular pressure, and macular thickness in patients with chronic kidney disease. Clin. Ophthalmol..

[15] Kiel J.W. (1999). Modulation of choroidal autoregulation in the rabbit. Exp. Eye Res..

[16] Chang I.B., Lee J.H., Kim J.S. (2017). Changes in Choroidal Thickness in and Outside the Macula After Hemodialysis in Patients with End-Stage Renal Disease. Retina.

[17] Ulaş F., Doğan Ü., Keleş A., Ertilav M., Tekçe H., Çelebi S. (2013). Evaluation of choroidal and retinal thickness measurements using optical coherence tomography in non-diabetic haemodialysis patients. Int. Ophthalmol..

[18] Ishibazawa A., Nagaoka T., Minami Y., Kitahara M., Yamashita T., Yoshida A. (2015). Choroidal thickness evaluation before and after hemodialysis in patients with and without diabetes. Investig. Ophthalmol. Vis. Sci..

[19] Tokuyama T., Ikeda T., Sato K. (2000). Effects of haemodialysis on diabetic macular leakage. Br. J. Ophthalmol..

[20] Bill A., Tornquist P., Alm A. (1980). Permeability of the intraocular blood vessels. Trans. Ophthalmol. Soc..

[21] Kur J., Newman E.A., Chan-Ling T. (2012). Cellular and physiological mechanisms underlying blood flow regulation in the retina and choroid in health and disease. Prog. Retin. Eye Res..

[22] Dolar-szczasny J., Flieger J., Kowalska B., Majerek D., Tatarczak-Michalewska M., Zakrocka I., Załuska W., Rejdak R. (2021). Hemodialysis effect on the composition of the eye fluid of cataract patients. J. Clin. Med..

[23] Nagaoka T., Takeyama Y., Kanagawa S., Sakagami K., Mori F., Yoshida A. (2004). Effect of haemodialysis on retinal circulation in patients with end stage renal disease. Br. J. Ophthalmol..

[24] Tosun Ö., Davutluoglu B., Arda K., Boran M., Yarangumeli A., Kurt A., Ozkan D. (2007). Determination of the effect of a single hemodialysis session on retrobulbar blood hemodynamics by color doppler ultrasonography. Acta Radiol..

[25] Zeng Z., Zhang Y. (2024). Improvement in Central Serous Chorioretinopathy in Renal Transplant Recipients Following Hemodialysis: Case Report. Transpl. Proc..

[26] Puro D.G. (2012). Retinovascular physiology and pathophysiology: New experimental approach/new insights. Prog. Retin. Eye Res..

[27] Törnquist P., Alm A., Bill A. (1990). Permeability of ocular vessels and transport across the blood-retinal-barrier. Eye.

[28] Shin Y.U., Lee D.E., Kang M.H., Seong M., Yi J.-H., Han S.-W., Cho H. (2018). Optical coherence tomography angiography analysis of changes in the retina and the choroid after haemodialysis. Sci. Rep..

[29] Shoshtari F.S., Biranvand S., Rezaei L., Salari N., Aghaei N. (2021). The impact of hemodialysis on retinal and choroidal thickness in patients with chronic renal failure. Int. Ophthalmol..

[30] Sun G., Hao R., Zhang L., Shi X., Hei K., Dong L., Wei F., Jiang A., Li B., Li X. (2019). The effect of hemodialysis on ocular changes in patients with the end-stage renal disease. Ren. Fail..

[31] Yang S.J., Han Y.H., Song G.I., Lee C.H., Sohn S.W. (2013). Changes of choroidal thickness, intraocular pressure and other optical coherence tomographic parameters after haemodialysis. Clin. Exp. Optom..

[32] Zegrari S., Mouallem A., Audard V., Jouan N., Grimbert P., Jung C., Sakhi H., Souied E.H., Miere A. (2023). Optical coherence tomography angiography analysis of changes in the retina and the choroid after hemodialysis for end stage kidney disease. Int. Ophthalmol..

[33] Wang L., Yin G., Yu Z., Chen N., Wang D. (2018). Effect of Hemodialysis on Eye Coats, Axial Length, and Ocular Perfusion Pressure in Patients with Chronic Renal Failure. J. Ophthalmol..

[34] Zhang Y., Weng H., Li Q., Wang Z. (2018). Changes in retina and choroid after haemodialysis assessed using optical coherence tomography angiography. Clin. Exp. Optom..

[35] Çelikay O., Cąlşkan S., Biçer T., Kabataş N., Gurdal C. (2015). The Acute Effect of Hemodialysis on Choroidal Thickness. J. Ophthalmol..

[36] Coppolino G., Bolignano D., Presta P., Ferrari F.F., Lionetti G., Borselli M., Randazzo G., Andreucci M., Bonelli A., Errante A. (2022). Acquisition of optical coherence tomography angiography metrics during hemodialysis procedures: A pilot study. Front. Med..

[37] Elbay A., Altinisik M., Dincyildiz A., Kutluturk I., Canan J., Akkan U., Koytak A., Ozdemir H. (2017). Are the effects of hemodialysis on ocular parameters similar during and after a hemodialysis session?. Arq. Bras. Oftalmol..

[38] Jung J.W., Chin H.S., Lee D.H., Yoon M.H., Kim N.R. (2014). Changes in subfoveal choroidal thickness and choroidal extravascular density by spectral domain optical coherence tomography after haemodialysis: A pilot study. Br. J. Ophthalmol..

[39] Chen H., Zhang X., Shen X. (2018). Ocular changes during hemodialysis in patients with end-stage renal disease. BMC Ophthalmol..

[40] Kang H.M., Choi J.H., Kim S.J., Moon S.J., Kim C.H. (2017). Changes in Choroidal Thickness after Hemodialysis and the Influence of Diabetes. J. Retin..

[41] Kal A., Kal O., Eroglu F.C., Oner O., Kucukerdonmez C., Yilmaz G. (2016). Evaluation of choroidal and retinal thickness measurements in adult hemodialysis patients using spectral-domain optical coherence tomography. Arq. Bras. Oftalmol..

[42] Nakano H., Hasebe H., Murakami K., Cho H., Kondo D., Iino N., Fukuchi T. (2020). Choroid structure analysis following initiation of hemodialysis by using swept-source optical coherence tomography in patients with and without diabetes. PLoS ONE.

[43] Chang Y.S., Weng S.F., Wang J.J., Jan R.L. (2019). Increased risk of central serous chorioretinopathy following end-stage renal disease. Medicine.

[44] Jonas J.B., Wang Y.X., Wei W.B., Xu J., You Q.S., Xu L. (2017). Chronic Kidney Disease and Eye Diseases: The Beijing Eye Study. Ophthalmology.

[45] Scorolli L., Giardina D., Morara M., Corazza D., Meduri R.A. (2003). Bilateral serous retinal detachments following organ transplantation. Retina.

[46] Ge G., Zhang Y., Zhang Y., Xu Z., Zhang M. (2020). Corticosteroids usage and central serous chorioretinopathy: A meta-analysis. Graefe’s Arch. Clin. Exp. Ophthalmol..

[47] Haimovici R., Koh S., Gagnon D.R., Lehrfeld T., Wellik S. (2004). Risk factors for central serous chorioretinopathy: A case-control study. Ophthalmology.

[48] Daruich A., Matet A., Dirani A., Bousquet E., Zhao M., Farman N., Jaisser F., Behar-Cohen F. (2015). Central serous chorioretinopathy: Recent findings and new physiopathology hypothesis. Prog. Retin. Eye Res..

[49] Wakakura M., Song E., Ishikawa S. (1997). Corticosteroid-induced central serous chorioretinopathy. Jpn. J. Ophthalmol..

[50] Polak B.C.P., Baarsma G.S., Snyers B. (1995). Diffuse retinal pigment epitheliopathy complicating systemic corticosteroid treatment. Br. J. Ophthalmol..

[51] Fawzi A.A., Holland G.N., Kreiger A.E., Heckenlively J.R., Arroyo J.G., Cunningham E.T. (2006). Central Serous Chorioretinopathy after Solid Organ Transplantation. Ophthalmology.

[52] Friberg T.R., Eller A.W. (1990). Serous retinal detachment resembling central serous chorioretinopathy following organ transplantation. Graefe’s Arch. Clin. Exp. Ophthalmol..

[53] Denic A., Rule A.D., Gaillard F. (2022). Kidney glomerular filtration rate plasticity after transplantation. Clin. Kidney J..

